# Odontoid fractures: A retrospective analysis of 53 cases

**DOI:** 10.4103/0019-5413.55975

**Published:** 2009

**Authors:** Arjun Shetty, Abhishek R Kini, Jagadish Prabhu

**Affiliations:** Department of Neurosurgery, Yenopoya Medical College and Neurosurgeon, Tejasvini Hospital, Mangalore - 575 002, India; 2 Department of Orthopaedics and Traumatology, Tejasvini Hospital and SSIOT, Kadri, Mangalore - 575 002, India

**Keywords:** Atlantoaxial dislocation, cervical spine injuries, odontoid fractures

## Abstract

**Background:**

The management of odontoid fracture has evolved but controversy persists as to the best method for Type II odontoid fractures with or without atlantoaxial (AA) instability. The anterior odontoid screw fixation can be associated with significant morbidity while delayed odontoid screw fixation has shown to be associated with reasonable good fusion rates. We conducted a retrospective analysis to evaluate the outcome of a trial of conservative management in type II odontoid fractures without atlantoaxial instability (Group A) followed by delayed odontoid screw fixation in cases in which fusion was not achieved by conservative treatment. The outcome of type II odontoid fracture with AA subluxation (Group B) was also analysed where closed reduction on traction could be achieved and in those atlantoaxial subluxations that were irreducible an intraoperative reduction was done.

**Materials and Methods:**

A retrospective evaluation of 53 cases of odontoid fractures treated over a 9-year period is being reported. All odontoid fractures without AA instability (n=29) were initially managed conservatively. Three patients who did not achieve union with conservative management were treated with delayed anterior screw fixation. Twenty-four cases of odontoid fractures were associated with AA instability; 17 of them could be reduced with skeletal traction and were managed with posterior fusion and fixation. Of the seven cases that were irreducible, the initial three cases were treated by odontoid excision followed by posterior fusion and fixation; however, in the later four cases, intra operative reduction was achieved by a manipulation procedure, and posterior fusion and fixation was performed.

**Results:**

Twenty-six of 29 cases of odontoid fracture without AA instability achieved fracture union with conservative management whereas the remaining three patients achieved union following delayed anterior odontoid screw fixation. 17 out of 24 odontoid fracture with atlantoaxial dislocation could be reduced on traction and these patients underwent posterior fusion and fixation. Optimal or near optimal reduction was achieved by on table manipulation in four cases which were irreducible with skeletal traction. Atlantoaxial stability was achieved in all cases. All cases were noted to be stable on evaluation with x-rays at six months.

**Conclusions:**

The initial conservative management and use of odontoid screw fixation only in cases where conservative management for 6–12 weeks has failed to provide fracture union have shown good outcome in type II odontoid fracture without AA instability rates. Intraoperative manipulation and reduction in patients where AA subluxation failed to reduce on skeletal traction followed by posterior fusion obviates the need for transoral odontoid excision.

## INTRODUCTION

Odontoid fractures constitute 10–20% of cervical spine fractures.^1^ They have been classified into three types^1^ by Anderson and d'Alonso.^2^ Type 1 (fractures through the tip of the dens) and type 3 (fractures that extend into the body of the C2 vertebra) tend to heal well with external immobilization (almost 100% in type 1 and 84–88% in type 3).^1^ However, these fractures when associated with atlanto axial instability need surgical intervention.

Type 2 fractures occur at the junction of the odontoid with the C2 body. These fractures tend to heal poorly with external immobilization (25–40% non-union).^1^ Type 2 fractures are further classified by Apfelbaum into three types based on the direction of the fracture line. Type 2a, where the line is anterior oblique; type 2b, where the line is posterior oblique; type 2c, where the line is horizontal.^3^ Patients with type 2a fracture have a poorer rate of union with external immobilization. Separation of more than 6 mm and fractures in elderly patients are also associated with poorer union rates.^4^,^5^

In patients subjected to odontoid fixation, fusion rates were found to be better in cases operated early compared with cases where surgery was delayed (88% in cases operated within 6 months and only 25% in cases operated after 18 months).^1^ The management of odontoid fractures is influenced by the type of fracture, the age of the patient, and the associated atlantoaxial instability.^6^ The presence of congenital vertebral bone anomalies and anomalies of the vertebral artery may further influence the surgical options available in a particular patient.

We conducted a retrospective analysis to evaluate the outcome of a trial of conservative management in type II odontoid fractures without atlantoaxial instability (Group A) followed by delayed odontoid screw fixation in cases in which fusion was not achieved by conservative treatment. The outcome of type II odontoid fracture with AA subluxation (Group B) was also analysed where closed reduction on traction could be achieved and in those atlantoaxial subluxations that were irreducible, an intraoperative reduction was done.

## MATERIALS AND METHODS

A retrospective analysis of 53 patients with odontoid fractures treated over a 9-year period was carried out. All patients seen in casualty with suspected cervical spine injuries, restriction of neck movements, or a neurological deficit suggestive of cervical cord injury and patients with concussional head injury were subjected to X-ray analysis of the cervical spine (anterior-posterior and lateral x-rays, further followed by flexion and extension lateral view and open mouth AP view). All patients with neurological deficits were mandatorily subjected to magnetic resonance imaging (MRI) scans, while patients with no neural deficit were subjected to MRI scans if the X-ray showed evidence of fracture instability.

Patients who were noted to have associated congenital anomalies as well as all cases where the anterior odontoid screw fixation or C1 lateral mass-C2 transpedicular stabilization was considered were also subjected to computed tomography (CT) scans to assess odontoid separation, the feasibility of placement of the screws, and the proximity to the foramen transversarium.^7^ CT angiogram was performed in cases where C1-C2 intraarticular fixation or C1 lateral mass-C2 transpedicular screw was used to assess the vertebral artery.

The time interval between the traumatic event and the patient presenting at our casualty varied from 2 hours to 16 days (avg. 3.62 days) and the patient's age varied from 10 to 63 years (avg. 33.03 years). The 53 cases were classified as per Anderson and d'Alonso^2^ into Type I (n=3), Type II (n=37), and Type III (n=13). Type II cases were further divided into Type a (n=12), Type b (n=17), and Type c (n=8) of Apfelbaum.^3^ These are further divided into group A and group B depending on the presence or absence of atlantoaxial stability [Table 1].

**Table 1:** Classification of cases according to Anderson and d'Alonso^2^, Apfelbaum^3^

	Type I	Type IIa	Type IIb	Type IIc	Type III	Total
Group A (without atlantoaxial instability)	3	5	10	4	7	29
Group B (with atlantoaxial instability)	0	7	7	4	6	24
Total	3	12	17	8	13	53

The patients were assessed for atlantoaxial instability using lateral flexion–extension films and open mouth X-rays.^6^ X-ray lateral flexion and extension films were used to asses instability indicated by a atlanto-dens interval (ADI) of more than 3 mm or a posterior atlanto-dens interval (PADI) of less than 19 mm. X-rays of the open mouth view were used to asses lateral mass overhang and to look for overlapping of the facets (winking sign),^7^ which would indicate a rotatory subluxation.

Twenty-nine patients had odontoid fractures without atlantoaxial subluxation (Type I, three cases; Type II, 19 cases; Type III, seven cases). All cases were initially subjected to external immobilization with a Halo brace for a period of 6 weeks and later if required odontoid fixation was done.

Twenty-four patients had odontoid fractures associated with atlantoaxial instability. All patients were subjected to skeletal traction for up to 72 h. In seven patients, the reduction could not be achieved despite 72 h of skeletal traction. The remaining 17 cases were found to be reducible. Due to the initial lack of familiarity with transarticular and lateral mass fixation techniques, patients with intact C1 posterior arch were fixed with sublamilar wires (n=6) and those with fractured or hypoplastic C1 arch underwent occipitocervical fixation (n=3). Subsequently transarticular fixation was performed in cases where reduction could be achieved in military tuck position and there was no high riding vertebral artery (n=4); the others underwent lateral mass fixation (n=3).

Of the seven cases of irreducible subluxation, the initial three patients underwent transoral odontoid excision followed by posterior C1-2 fusion with sublaminar wiring. All these cases were performed as two-stage procedures.

The last four cases in whom atlantoaxial subluxation was irreducible with skeletal traction were subjected to intraoperative manipulation of C1-C2 to achieve reduction, after which posterior fixation was performed, obviating the need for transoral odontoid excision.

### Reduction procedure

**Figure 1:**
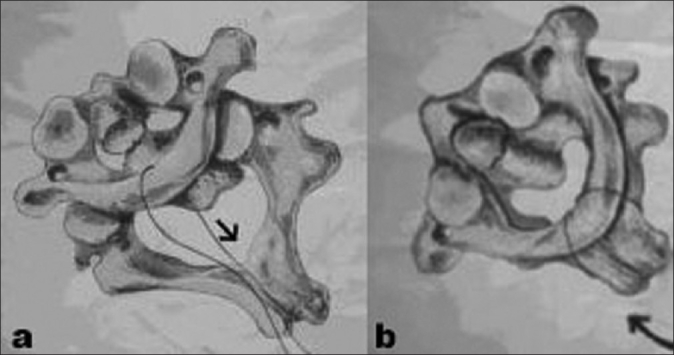
(a) Photograph shows C1 spine pulled downward and backwards. (b) C2 spine pushed upward and forward

C1C2 was approached by posterior midline approach in prone position. One loop of 21 gauge stainless steel was passed under the C1 lamina while a second loop was passed under C1-C2 laminae. After excision of the C2 spinal ganglion, the capsule of the C1-C2 articular facets and articular cartilage are drilled and curetted to free the joints. Steady traction was applied downwards and backwards on the C1 posterior arch using the sublaminar C1 wire; at the same time the C2 spinous process was pushed forward and upward under constant fluoroscopic guidance [Figure 1]. Once acceptable reduction was achieved, the C1-C2 sublaminar wire was tightened over an iliac bone graft to achieve fixation and the C1 wire was removed. Subsequently, C1-C2 fixation was performed using transarticular screws or lateral mass fixation using screws and plates (two cases each).

All patients were subjected to detailed neurological evaluation as well as cervical spine radiographs, open mouth view and lateral flexion and extension views at the time of discharge. They were reviewed at 6-week, 12-week, and 6-month intervals and on each of these visits, neurological assessment and X-rays were recorded. Fusion of odontoid fractures and assessment of post-fixation stability were confirmed by radiographs (open mouth view, flexion, and extension lateral films).^8^ The minimum follow up was six months.

## RESULTS

Out of twenty nine patients, all three cases of Type I fractures (100%) and six of the Type III fractures (85.7%) showed evidence of union after 6 weeks of immobilization, while seventh patient achieved union after being subjected to an additional 6 weeks of halo immobilizations.

Of the 19 cases (out of 29 patients) of type II odontoid fractures without atlantoaxial instability, 13 patients achieved union following 6 weeks of immobilization. Of the remaining six patients, three patients (fracture site separation less than 6 mm) achieved union after being subjected to an additional 6 weeks of immobilization. Hence, 16 of 19 (84.2%) patients achieved fusion with halo brace immobilization.

**Figure 2:**
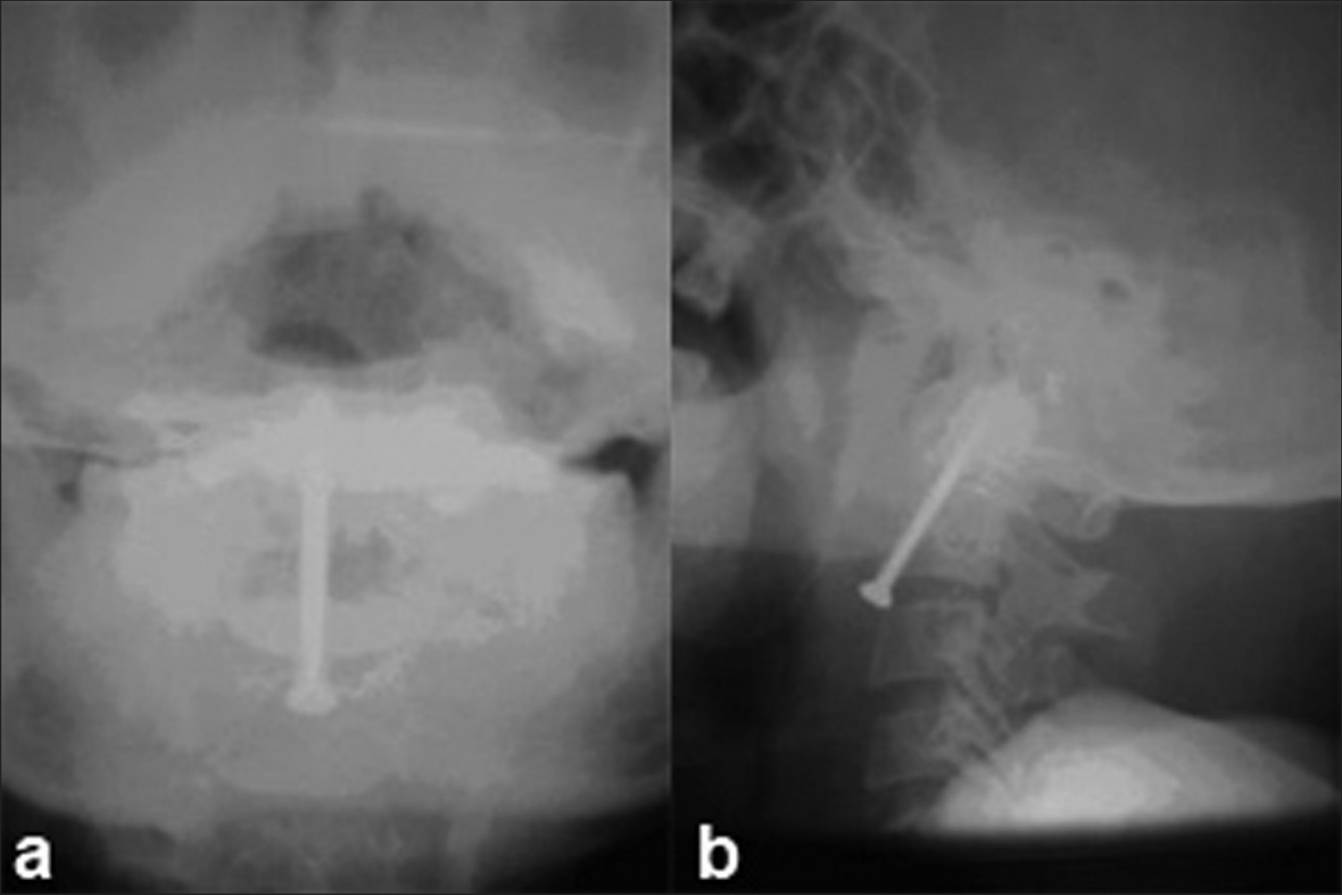
Anteroposterior (a) and lateral view (b) of cervical spine shows odontoid fixation with a single cortical screw. The screw is placed up to the cortex but is not traversing it

Two cases where fusion was not achieved after 6 weeks and had a fracture site separation of more than 6 mm were subjected to anterior odontoid screw fixation. One patient who had a less than 6 mm fracture separation and did not achieve fusion despite 12 weeks of immobilization also underwent anterior odontoid screw fixation. Thus overall three patients needed anterior adontoid screw fixation. All three patients subjected to odontoid screw fixation achieved union. In all cases, a single cortical screw was used and lag effect was achieved by oversize tapping of the proximal bone [Figure 2].

**Chart 1:**
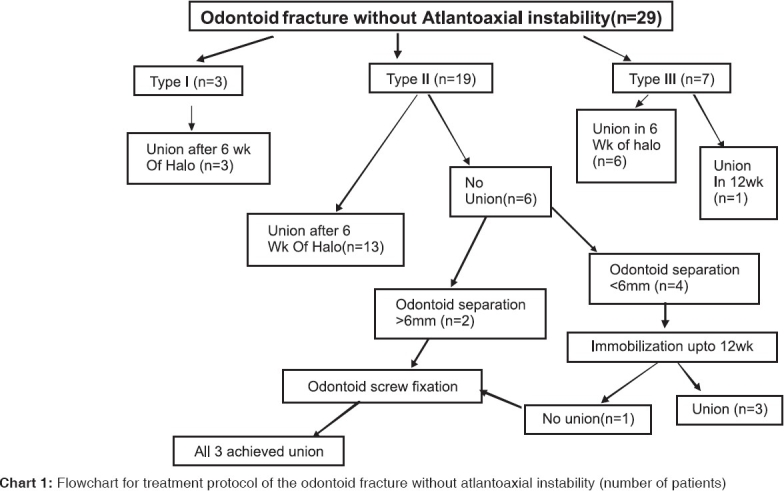
Flowchart for treatment protocol of the odontoid fracture without atlantoaxial instability (number of patients)

**Figure 3:**
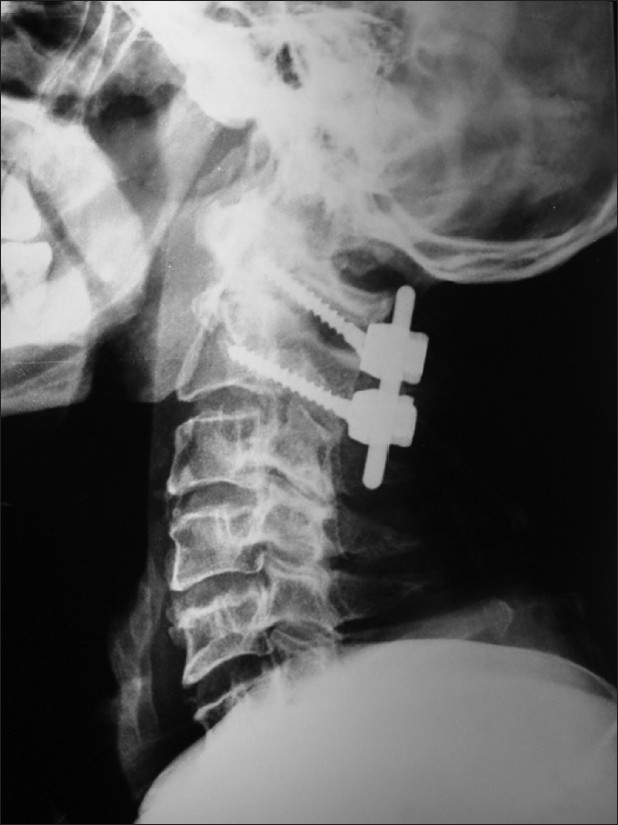
X-ray lateral view of cervical spine shows C1 lateral mass and C2 transpedicular fixation with polyaxial screws and rods with transarticular and interlaminar bone graft in a patient in group B

One patient had persistent cervical pain and restriction of neck movements. No other morbidity was associated with this procedure [Chart 1]. Twenty-four patients had odontoid fracture with atlantoaxial instability. Seven of them were irreducible while in 17 reduction could be achieved. Of the seven cases of odontoid fracture with irreducible atlantoaxial instability, the initial three cases underwent an initial transoral odontoid exicision followed by a second stage C1-C2 sublamilar wiring with Gallie fusion. Revision and transarticular fixation was needed in one patient. In the remaining four cases, on-table reduction by direct C1-C2 manipulation was performed to achieve complete reduction in three cases and near total reduction in one case. In all four cases, posterior fixation (transarticular fixation in two cases and C1 lateral mass-C2 transpedicular fixation in two cases [Figure 3]) was performed. Reduction was optimal in 3 cases and near optimal in one case. All cases were stable on flexion extension views at six months.

Six patients with reducible atlantoaxial instability were treated with sublaminar wiring and Gallie fusion. In two cases with C1 posterior arch fractures and one case with partly occipitalized hypoplastic C1 arch, occipito-C2 fusion was achieved using plates and screws with supplemented onlay graft. Significant restriction of neck movement was noted in both cases; however, the X-ray did not show any evidence of instability on follow-up.

Of the nine cases that were treated with C1-C2 sublaminar wires and Gallie fusion, three cases showed evidence of graft failure and loosening of wires (33%). In all three cases, fresh wiring with supplementation of graft with transarticular screw fixation was performed.

Transarticular screw fixation was performed in a total of eight cases. In five patients, the fixation was supplemented with sublaminar wires and Gallie fusion. In two cases, intraarticular fusion was performed. In one case, the transarticular fusion was supplemented by an occipitocervical fixation and onlay graft. All cases were stable on flexion extension views at six months. Neck movement was significantly restricted in a patient in whom occipitocervical fixation was done.

**Figure 4:**
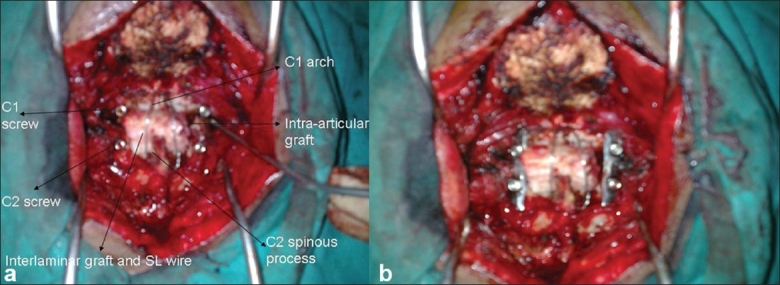
Operative picture (a) showing C1 lateral mass and C2 transpedicular screws with interlaminar graft, sublaminar wires and intraarticular graft. Operative picture (b) showing plate and screws in position

All cases treated with transarticular screws with C1 lateral mass-C2 transpedicular fixation [Figures 3 and 4] showed no evidence of instability on follow-up flexion extension radiographs. Studies have reported success rates of 85–100% in both transarticular fixation and in C1-C2 lateral mass fixation procedures.

One patient who underwent C1 lateral mass-C2 transpedicular fixation sustained a vertebral artery injury during placement of C2 transpedicular screw. Hemostasis was achieved with bone wax. As the screw was already placed on the opposite side, fixation on the side of vertebral artery injury was deffered. One patient developed an occipital bed sore that needed a small rotation flap.

**Chart 2:**
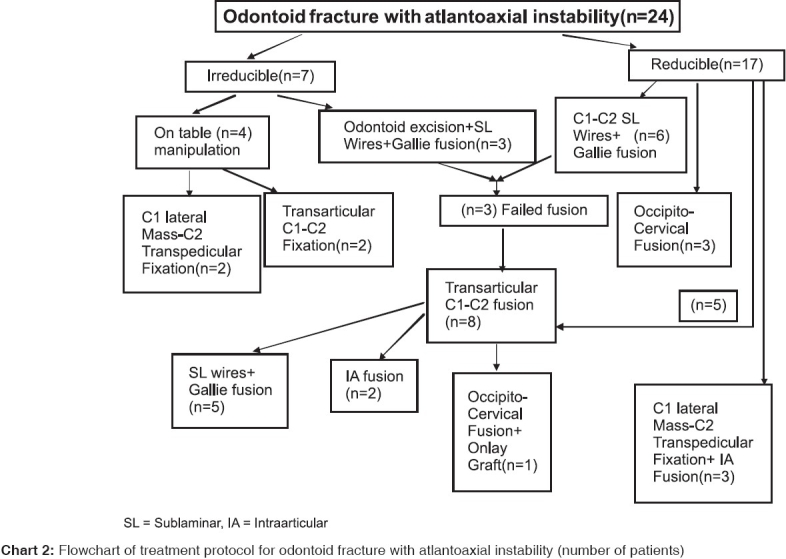
Flowchart of treatment protocol for odontoid fracture with atlantoaxial instability (number of patients)

There was no mortality encountered in the study and the complications associated with the procedures performed are enlisted in Table 2, Chart 2.

**Table 2:** Complications associated with various surgical procedures

Procedure	No.	Graft failure	Injury to vertebra	Wound infection	Pneumonia	DVT	Suboptimal implant	Occipital bedsore
Transoral odontoid excision	3	-	-	-	1	1	-	-
C1-C2 SL wiring + Gallie fusion	6 + 3	3	-	-	-	-	-	-
C1-C2 transarticular screws + SL wiring + fusion	2 + 3	-	-	-	-	-	1	-
C1-C2 transarticular screws + IA fusion	2	-	-	1	-	-	-	-
C1-C2 transarticular screws with occipito-cervical fixation	1	-	-	-	-	-	-	-
C1-C2 lateral mass transpedicular fixation + IA fusion	3	-	1	1	-	-	-	1
Anterior odontoid screw fixation	3	-	-	-	-	-	-	-
Occipitocervical fixation	3	-	-	-	-	-	-	-

SL = Sublamilar; IA = Intraarticular

## DISCUSSION

Acute stable type II odontoid fractures have been treated with anterior odontoid fixation with exellent results. However the morbidity and mortality associated with the procedure especially in the elderly and the good results achieved with delayed odontoid fixation prompts us to consider a trial of conservative management in these patients and reserving odontoid fixation for patients in whom conservative treatment has failed. Of the 29 cases of odontoid fractures without subluxation, the fusion rates achieved by us are in keeping touch with fusion rates achieved in earlier studies (100% in Type I and 84–88% in Type III fractures).^1^

Type II fractures respond variably to conservative treatment with a halo brace and failure of fusion has been reported in 21–45% of the cases.^10^–^13^ Type II fractures without atlantoaxial instability when treated with anterior odontoid screw fixation have been associated with fusion rates of 90–95%.^14^,^15^ Management of acute Type II fractures without atlantoaxial subluxation directly with odontoid screw fixation has been advocated in many series, voicing concern about the possible risk of non-union in cases where fixation has been delayed; however, Apfelbaum *et al*.^1^ have reported fusion in 83% of the cases fixed with anterior screws up to 6 months after injury. Other studies have also shown good results with delayed odontoid screw fixation.^16^ Anterior screw fixation is not without complications and Platzer *et al*.^14^ have reported on overall mortality of 4% (and as high as 9% in geriatric cases). Other studies have also shown significant mortality and morbidity.^17^–^19^

We have given a trial of conservative treatment to all patients with odontoid fractures without atlantoaxial instability (n=19). Only those patients who did not benefit from immobilization with a halo brace underwent odontoid screw fixation (n=3). We have been able to achieve fracture union in 16 of 19 (84%) Type II fracture without AA instability while in 3 cases delayed anterior odontoid screw fixation was done to achieve union. Thus 3 of 19 (type II) only required anterior odontoid screw fixation.

The placement of two odontoid screws has been advocated by some surgeons to reduce the rotational load on a single screw. However, patients treated with single- and double-screw constructs have had similar results.^1^,^20^ We have not used cannulated lag screws and specialized tube systems due to lack of availability at our center. We also prefer to pass the screw up to the tip of the odontoid but did not traverse the cortex as advocated by Apfelbaum^1^ [Figure 2].

Patients with odontoid fractures associated with atlantoaxial instability are initially subjected to skeletal traction. Cases which are reducible on traction are treated by a posterior fixation procedure. Both transarticular screw fixation as well as lateral mass fixation provide excellent results.

Our initial three cases of irreducible odontoid fractures with subluxation were treated with transoral odontoid excision followed by a Gallie fusion; however, in the four cases treated in the last 3 years, atlantoaxial joint capsule and articular cartilage removal along with manipulations as discussed earlier allowed adequate reduction. This technique is a variation of the technique described by Goel *et al*. for managing fixed atlantoaxial subluxation.^21^,^22^ Various methods of manipulation to achieve reduction and alignment of odontoid fractures as well as atlantoaxial subluxation have been advocated. Intraoperative transoral manipulation,^23^ skeletal traction manipulation, and direct manipulation of the C1-C2 posterior elements have been recommended.

We have found good results with direct manipulation of C1-C2 posterior elements as described earlier and feel this would enable most fresh cases of atlantoaxial subluxation to be managed without transoral odontoid excision. We have not attempted to directly distract the joint space, which is a more demanding technique. While our method allows adequate reduction of atlantoaxial subluxations, correction of basilar invagination could possibly need distraction of the joints and the use of a spacer or graft, as advocated by Goel.^21^,^22^

Both C1-C2 transarticular screw fixation as well as C1 lateral mass-C2 transpedicular fixation provided significantly better stability as compared with C1-C2 sublaminar wiring with Gallie fusion (85–100% success rates have been reported with both procedures).^8^,^24^,^25^

In centers where instrumentation for percutaneous transarticular fixation is not available, open transarticular C1-C2 fixation can only be performed where complete reduction of the subluxation can be achieved in a “military tuck” position to allow the passage of screws in the correct angle. Transarticular fixation is also avoided in cases where pre-operative assessment reveals a high riding vertebral artery, the incidence of which may be as high as 20% of the cases.^24^–^26^

**Figure 5:**
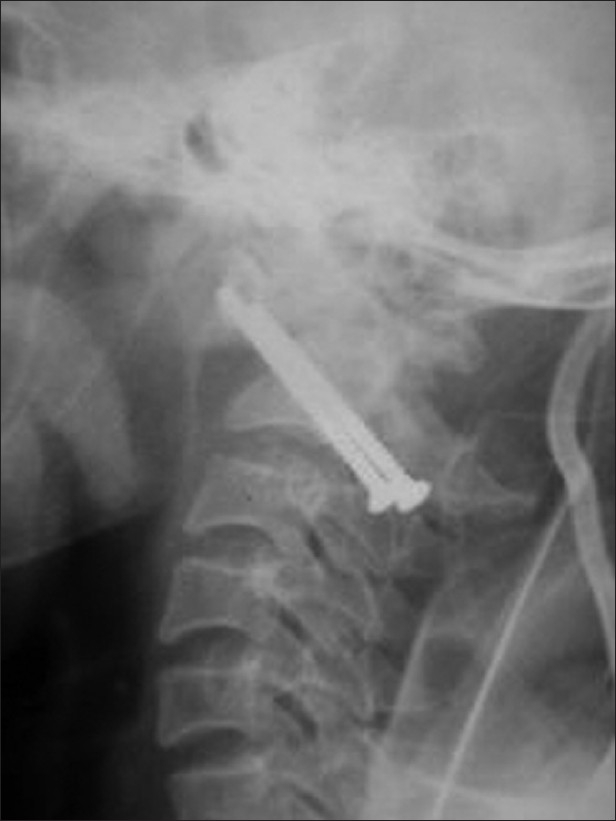
X-ray cervical spine lateral view showing transarticular C1 C2 fixation. The suboptimal reduction cannot be corrected after passing of the transarticular screw unlike in the lateral mass fixation where further reduction can be achieved by tightening the screws over the rods

Another problem associated with transarticular fixation is that once the screw has been passed in a position of suboptimal reduction, further correction is not possible. In one of our patients, the reduction was noted to be suboptimal after passage of transarticular screws; however further correction could not be done unlike in cases treated with lateral mass fixation where further reduction is possible while tightening the screws over the rods [Figure 5].

In cases where reduction cannot be achieved in a “military tuck” position, in patients with high riding vertebral artery, and in obese patients with short neck, the C1 lateral mass-C2 transpedicular fixation using lateral screws and plates or rods should be considered. In cases where reduction was suboptimal before fixation, further correction is noted after tightening of screws. This correction may lead to previously applied sublaminar wires becoming loose, necessitating retightening of the wires after plating.

Vertebral artery injury is a matter of concern in both the procedures. The artery can be injured at the upper border of the C1 arch while placing the C1 lateral mass screws and can also be injured as it loops out of the C2 foramen transversarum between C1 and C2 while placing the transarticular screws or the C2 transpedicular screws. Transarticular fixation is said to have a higher risk of vertebral artery injury. However, in our series, the only vertebral artery injury was in a child who underwent C1 lateral mass-C2 transpedicular fixation. As the opposite side screw had already been placed, fixation on the side of the injury was deferred, hemostasis was achieved by sealing the hole with bone wax, and the child did not suffer any problem due to injury to the vertebral artery.

In C1 lateral mass screw placement, the exposure of the lateral mass and the articular joint can be facilitated by excising or mobilizing the C1 ganglion. We routinely excise this ganglion as this allows excellent exposure of the articular joint and the lateral mass and aids in the placement of screws, decortication of the joint space, and placement of the extraarticular graft. However, we have found one patient to have developed an occipital bed sore, which may be attributed to the loss of sensations due to excision of the ganglion, thus making a strong case for attempting to preserve the ganglion in future cases.

Various authors have described differences in placing C1 lateral mass screws in children. However, we have not had difficulty passing C1 screws in two children (age 10/12 years) in whom lateral mass fixation was performed.

All cases of lateral mass transpedicular fixation were performed under microscopy, which we feel significantly reduces the venous bleeding in addition to reducing the difficulty of placing the C1 screws.

## CONCLUSION

Odontoid fracture without atlantoaxial subluxation showed successful union on conservative management with a halo brace The anterior odontoid fixation is required due to failure of conservative treatment.

In cases associated with atlantoaxial subluxation, most cases that are irreducible with skeletal traction are amenable to intraoperative manipulation obviating the need for odontoid excision in most cases.

Both C1-C2 transarticular fixation and C1 lateral mass-C2 transpedicular fixation provide excellent stability in odontoid fractures associated with atlantoaxial instability. The lateral mass technique is preferable in cases where pre-operative reduction is suboptimal and in patients with a high riding vertebral artery where transarticular fixation is best avoided.
